# Asymptomatic idiopathic intracranial hypertension: Prevalence and prognosis

**DOI:** 10.1111/ceo.14256

**Published:** 2023-05-27

**Authors:** Mark Thaller, Victoria Homer, Susan P. Mollan, Alexandra J. Sinclair

**Affiliations:** ^1^ Translational Brain Science, Institute of Metabolism and Systems Research University of Birmingham Birmingham UK; ^2^ Department of Neurology University Hospitals Birmingham NHS Foundation Trust Birmingham UK; ^3^ Centre for Endocrinology, Diabetes and Metabolism Birmingham Health Partners Birmingham UK; ^4^ Cancer Research (UK) Clinical Trials Unit University of Birmingham Birmingham UK; ^5^ Birmingham Neuro‐Ophthalmology University Hospitals Birmingham NHS Foundation Trust Birmingham UK

**Keywords:** asymptomatic, headache, incidental, pseudotumor cerebri, vision

## Abstract

**Background:**

Little is known about the presentation and prognosis of asymptomatic idiopathic intracranial hypertension (IIH). Papilloedema can be found incidentally on routine fundus examination, with many of these patients actually having symptoms on direct questioning. The aim was to evaluate visual and headache outcomes in people with IIH who present with or without symptoms.

**Methods:**

Prospective observational cohort study, between 2012 and 2021, 343 people with confirmed IIH diagnosis were enrolled in the IIH:Life database. Outcomes such as vision (LogMAR); Humphrey visual field perimetric mean deviation (PMD) and optical coherence tomography (OCT) and headache were evaluated using LOESS (locally weighted scatterplot smoothing) graphs and regression analysis.

**Results:**

One hundred and twenty‐one people had incidentally found papilloedema, with 36 people with completely asymptomatic presentations. Those with asymptomatic IIH at diagnosis had similar visual prognosis compared to those with symptomatic disease. Sixty‐six percent of the asymptomatic cohort became symptomatic during follow‐up, and of these the predominant symptom was headache (96%). Headache frequency during follow‐up was lower in the asymptomatic cohort.

**Conclusions:**

The prognosis of those with IIH who present with or without symptoms is similar.

## 1 INTRODUCTION

Papilloedema, swelling of both optic discs caused by raised intracranial pressure (ICP), is required for the definite diagnosis of Idiopathic Intracranial Hypertension (IIH).^1^, ^2^ Typically these patients present with headaches and visual disturbances,^3^, ^4^, ^5^, ^6^ however increasingly people are referred to ophthalmology and IIH clinics with incidental papilloedema following routine optician reviews or neurological examination,^7^ with many of these actually having symptoms on direct questioning.^8^

The precise prevalence of asymptomatic IIH is unclear from the literature. Children appear to have fewer symptoms than adults^9^, ^10^ but the previous literature has been based on small case series or case reports. Additionally, asymptomatic patients at baseline may not remain asymptomatic.^8^

Incidentally discovered papilloedema is more prevalent as a presenting feature of IIH in UK compared with US populations (48% vs. 30%; *p* < 0.001),^7^ however this may indicate different access to ophthalmological assessments rather than a true variation, and in that study the authors were unable to assess whether the patients were asymptomatic. As IIH predominantly affects reproductive‐aged women with obesity,^1^, ^4^, ^11^, ^12^, ^13^ screening for papilloedema in people with obesity has been suggested. However, this would not meet the criteria for an acceptable screening programme with one large bariatric surgery study finding that incidental papilloedema was rare (4/606, 0.66%).^14^

Theoretically, those with no symptoms may have a delay in their diagnosis, or silent relapses and therefore may have the potential for adverse longer‐term visual outcomes, as compared to those with symptoms. Alternatively, asymptomatic presentations may indicate that the disease begins prior to symptom development, therefore could benefit from earlier interventions. There is an absence of longitudinal outcome data in the asymptomatic subset of IIH and evaluation of their outcomes may guide management decisions. This study therefore aimed to evaluate the visual and headache outcomes for people diagnosed with asymptomatic IIH at presentation, and compare them to IIH patients who presented with symptoms.

## 2 METHODS

### 2.1 Ethics statement

This study conformed to the tenets of the Declaration of Helsinki. The study was ethically approved by National Health Service National Research Ethics Committee (14/LO/1208), IIH:LIFE study. Written informed consent was obtained from all participants.

### 2.2 Patients

Eligible patients were those who met the revised diagnostic criteria for IIH^2^ and had data recorded for IIH symptomatology at diagnosis (including ‘incidental papilloedema’). Patients with secondary causes of raised ICP and IIH without papilloedema were excluded.

### 2.3 Symptom status

Incidental papilloedema was defined as bilateral optic disc oedema which was found following routine examination most likely in the setting of a routine optician or optometry tests, and where the patient was subsequently diagnosed with IIH. Some of these patients on direct questioning in the hospital setting were not necessarily asymptomatic. Asymptomatic status was defined as a total absence of headache, transient visual obscurations (TVOs), pulsatile tinnitus and other visual symptoms. This was based on direct questioning of symptoms at their first clinical visit, focussing on the time when the optic disc swelling was detected.

### 2.4 Outcomes

Visual outcomes were the logarithm of the minimum angle of resolution (LogMAR) visual acuity, Humphrey visual field perimetric mean deviation (PMD) and optical coherence tomography (OCT) imaging measures (average global peripapillary retinal nerve fibre layer (RNFL), manually measured peripapillary total retinal thickness (TRT), and macular ganglion cell layer (GCL) volume). Headache outcomes were assessed by headache frequency (monthly headache days), migraine‐like headache frequency (monthly migraine‐like headache days), headache severity (0–10 numerical rating scale), and headache disability using the Headache Impact Test‐6 (HIT‐6).

### 2.5 Statistical analysis

The methods for data collection and statistical analysis have previously been reported.^4^ Statistical analysis was performed using R v4.1.0. Mean and standard deviations for continuous variables, and percentages for binary or categorical variables were used for summary statistics. Regression modelling used lme4^15^ with the creation of LOESS (locally weighted scatterplot smoothing) graphs prior to regression analysis to ascertain the relationship between variables and any emergent trends.

## 3 RESULTS

A total of 343 patients were included, and their baseline characteristics are reported in Table 1. The prevalence of incidental papilloedema in this cohort was 35% (121/343). Asymptomatic IIH at time of papilloedema recognition was found to be uncommon (10%, 36/343) (Table 1) and only 3.5% (12/343) remained asymptomatic during the follow‐up period.

**TABLE 1:** Baseline table by whether asymptomatic at diagnosis.

	All	Asymptomatic	Symptomatic
IIH patients (*n*)	343	36	307
Surgical intervention (*n*)	37	1	36
Females (*n*)	336	33	303
Age (SD) (years)	30.9 (9.0)	30.1 (9.8)	30.9 (8.9)
BMI (SD) (kg/m^2^)	38.4 (8.5)	35.8 (5.8)	38.7 (8.8)
Weight (SD) (kg)	101.2 (24.7)	99.1 (20.0)	101.4 (25.2)
Diagnostic lumbar puncture opening pressure (SD) (cmCSF)	34.8 (10.5)	36.3 (7.5)	34.6 (10.7)

Headaches were the most common symptom (90%, 307/343) in the cohort either at diagnosis or whilst being followed up. The other symptoms in descending frequency were tinnitus (40%, 137/343), visual disturbances (36%, 125/343) and transient visual obscurations (TVOs) (30%, 102/343). Only a third of those who were asymptomatic at baseline remained so during follow up (12/36, 33%). In those who became symptomatic, the most frequent symptom was headache (23/24, 96%), with other symptoms being less common (tinnitus (9/24, 38%), visual disturbances (2/24, 8%) and transient visual obscurations (1/24, 4%)).

Asymptomatic IIH patients had similar age, lower body mass index (BMI) and higher diagnostic lumbar puncture opening pressure (LP OP) than those with symptoms (Table 1), although the differences were not significant. The surgical intervention rate was lower in those with an asymptomatic presentation (asymptomatic 1/36, 2.8% compared with 36/307, 11.7% for symptomatic). The sole asymptomatic patient who underwent surgical management and became symptomatic 3 months after the initial review, had CSF diversion surgery 6 months after initial presentation. In the symptomatic group, those who required surgical intervention had a higher BMI [40.7 (8.8), vs. 38.4 (8.7) kg/m^2^] and higher diagnostic LP OP [37.7 (20.5), vs. 34.3 (9.0) cmCSF] compared with medically managed symptomatic patients, although neither was statistically significantly different.

A personal migraine history was present in 42% (15/36) of the asymptomatic group and 47% (145/307) of the symptomatic cohort. A family history of migraine was present in 28% (10/36) of the asymptomatic group and 22% (68/307) of the symptomatic group. A non‐statistically significant trend was seen in those patients who subsequently became symptomatic, with a previous migraine history in 46% (11/24) and family history in 33% (8/24) family history of migraines, which is in contrast to those remaining asymptomatic of 33% (4/12) and 17% (2/12), respectively.

Where patients had at least one follow‐up visit (*n* = 234), the median follow‐up (range) was 19.5 (1–87) months and this was longer for the asymptomatic subset [25 (1–72) months, *n* = 27] than symptomatic [19 (1–87) months].

### 3.1 Visual outcomes

**FIGURE 1:**
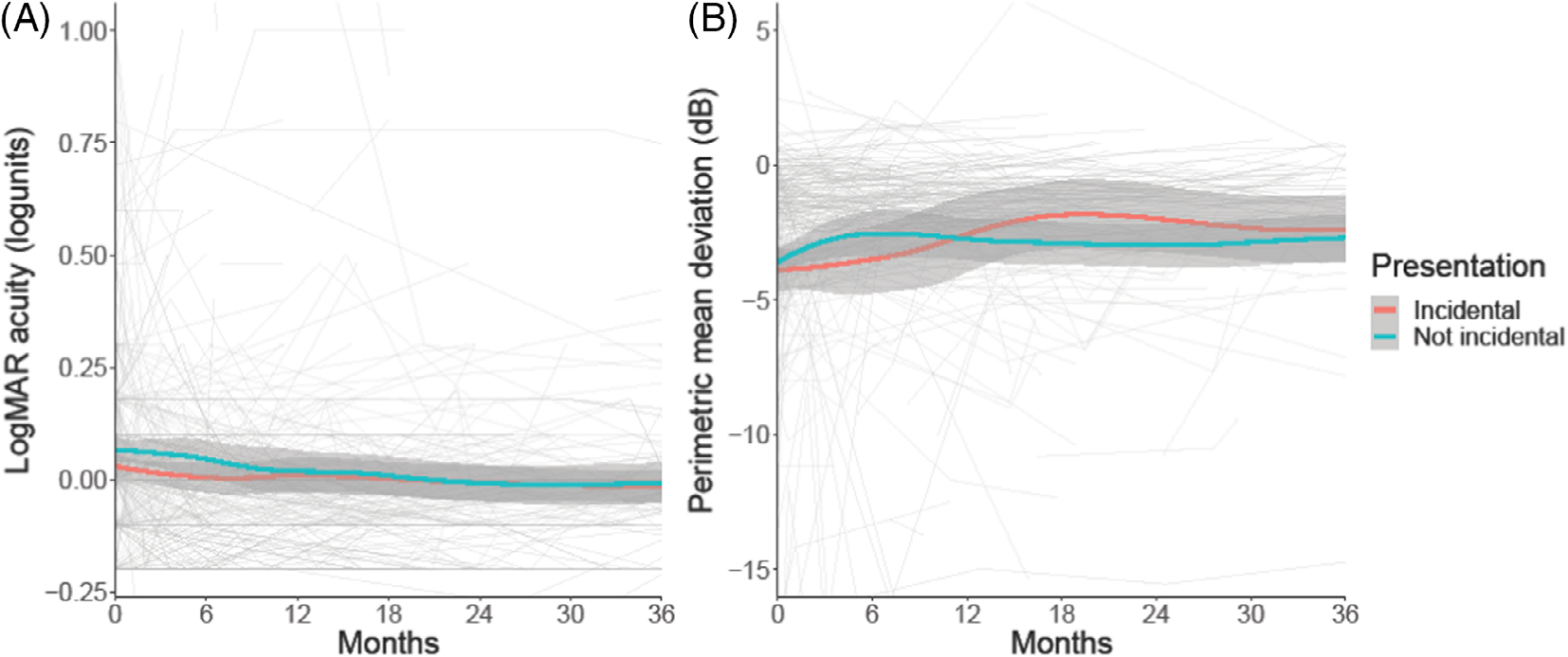
Longitudinal visual data categorised by symptoms at diagnosis, with Incidental papilloedema illustrated. LOESS smoothers added to show trends across the categories. (A) LogMAR visual acuity (logunits). (B) Perimetric mean deviation measured by Humphrey visual field 24–2 testing (dB)

**FIGURE 2:**
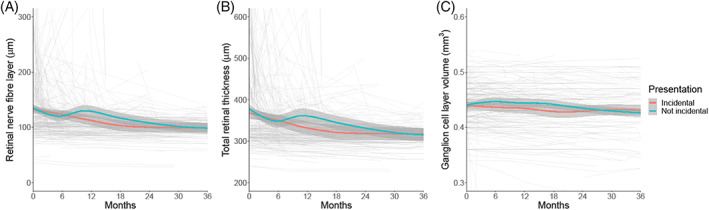
Longitudinal visual data categorised by symptoms at diagnosis, with Incidental papilloedema illustrated. LOESS smoothers added to show trends across the categories. (A) Retinal nerve fibre layer thickness measured on Optical Coherence Tomography (μm). (B) Total retinal thickness of optic nerve head measured on Optical Coherence Tomography (μm). (C) Macular ganglion cell layer volume measured on Optical Coherence Tomography (mm^3^).

Incidental papilloedema at diagnosis did not influence visual acuity or visual fields at baseline and over time their trajectories were similar with a slow improvement noted in both groups (Table 2, Figure 1A,B). OCT imaging measures of RNFL and TRT were slightly worse on average in the incidental cohort at baseline although this was not statistically significant (Table 2, Figure 2A,B). Both groups improved over time (Table 2). Macular ganglion cell layer volumes were comparable between the groups with a very slow deterioration in both groups over time (Table 2, Figure 2C).

**TABLE 2:** Baseline and trajectory for visual and headache outcomes according to incidental papilloedema at diagnosis calculated by regression modelling.

	Baseline estimate	Change per month
LogMAR visual acuity, logunits		
Incidental	−0.0049 (95% CI: −0.0594, 0.0495	−0.0005 logunits/month (95% CI: −0.0023, 0.0014)
Not incidental	0.0449 (95% CI: 0.0077, 0.0821)	−0.002 logunits/month (95% CI: −0.0034, −0.0005)
Humphrey visual field perimetric mean deviation, dB		
Incidental	−2.5491 (95% CI: −3.8485, −1.2496)	0.0544 dB/month (95% CI: −0.0328, 0.1416)
Not incidental	−3.6149 (95% CI: −4.4927, −2.737)	0.1254 dB/month (95% CI: 0.0599, 0.1909)
Global peripapillary retinal nerve fibre layer, μm		
Incidental	142.77 (95% CI: 125.55, 159.99)	−1.78 μm/month (95% CI: −2.76, −0.81)
Not incidental	133.4 (95% CI: 122.05, 144.75)	−1.45 μm/month (95% CI: −2.17, −0.72)
Global peripapillary total retinal thickness, μm		
Incidental	372.58 (95% CI: 348.79, 396.36)	−2.52 μm/month (95% CI: −3.65, −1.4)
Not incidental	360.2 (95% CI: 344.47, 375.93)	−2.33 μm/month (95% CI: −3.17, −1.49)
Macular ganglion cell layer volume, mm^3^		
Incidental	0.4412 (95% CI: 0.426, 0.4564)	−0.0006 mm^3^/month (95% CI: −0.001, −0.0003)
Not incidental	0.4439 (95% CI: 0.4339, 0.4539)	−0.0003 mm^3^/month (95% CI: −0.0005, 0)
Headache frequency (days/month)		
Incidental	17.93 (95% CI: 14.9, 20.96)	0.01 units/month (95% CI: −0.24, 0.26)
Not incidental	20.2 (95% CI: 17.87, 22.53)	−0.18 units/month (95% CI: −0.4, 0.04)
Migraine‐like headache frequency (days/month)		
Incidental	5.43 (95% CI: 2.58, 8.29)	0.21 units/month (95% CI: −0.03, 0.44)
Not incidental	10.06 (95% CI: 7.99, 12.12)	−0.15 units/month (95% CI: −0.35, 0.05)
Headache severity (0–10 scale)		
Incidental	6.0852 (95% CI: 5.2133, 6.957)	0.0333 units/month (95% CI: −0.0346, 0.1011)
Not incidental	6.7345 (95% CI: 6.1173, 7.3516)	−0.0372 units/month (95% CI: −0.0945, 0.0202)
HIT‐6 (score 36–78)		
Incidental	61.67 (95% CI: 58.09, 65.25)	0.15 units/month (95% CI: −0.06, 0.37)
Not incidental	60.86 (95% CI: 58.23, 63.48)	−0.05 units/month (95% CI: −0.22, 0.11)

**FIGURE 3:**
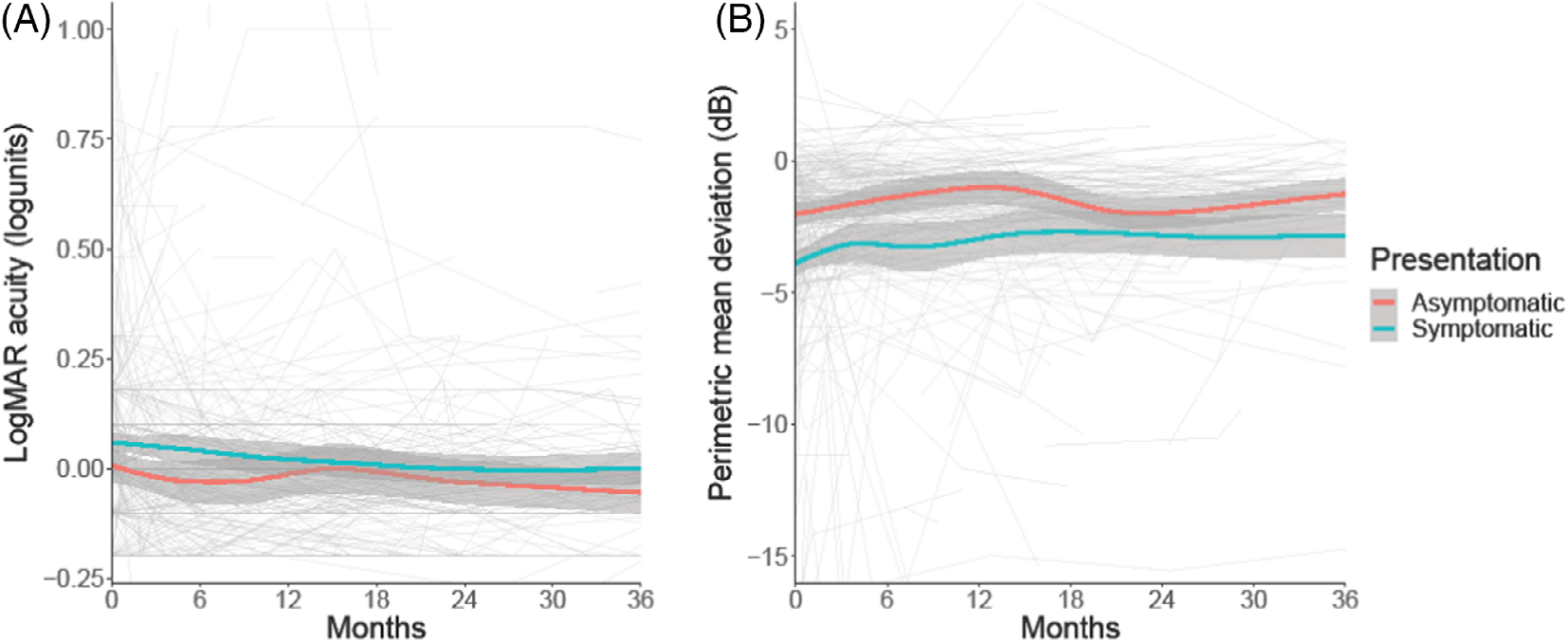
Longitudinal visual data categorised by symptoms at diagnosis, with Asymptomatic presentations illustrated. LOESS smoothers added to show trends across the categories. (A) LogMAR visual acuity (logunits). (B) Perimetric mean deviation measured by Humphrey visual field 24–2 testing (dB).

**FIGURE 4:**
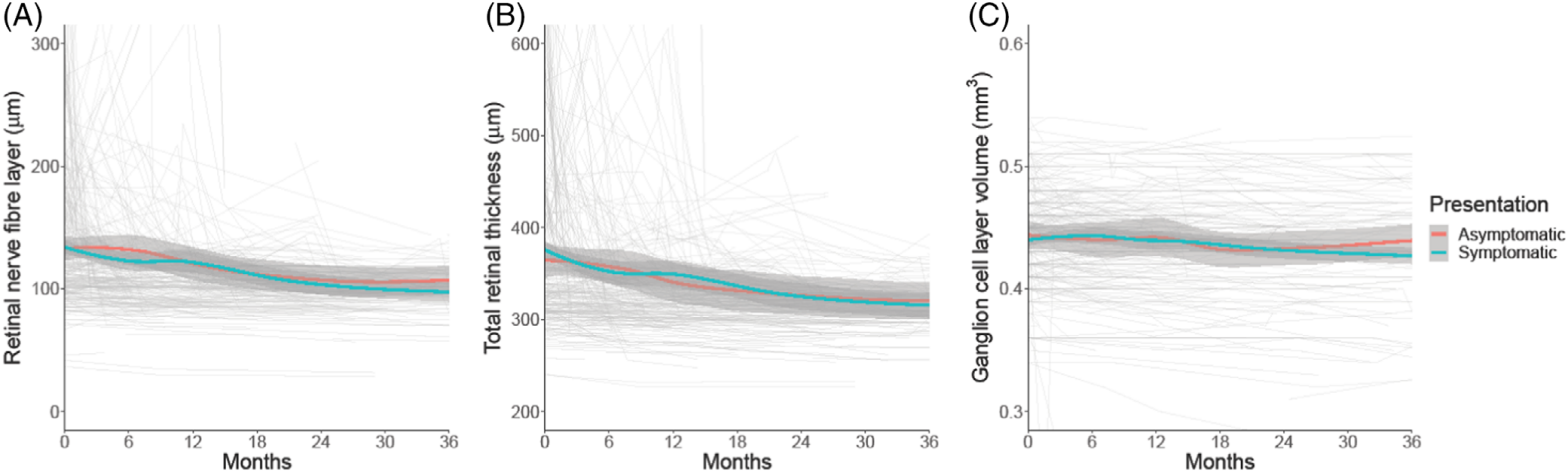
Longitudinal visual data categorised by symptoms at diagnosis, with Asymptomatic presentations illustrated. LOESS smoothers added to show trends across the categories. (A) Retinal nerve fibre layer thickness measured on Optical Coherence Tomography (μm). (B) Total retinal thickness of optic nerve head measured on Optical Coherence Tomography (μm). (C) Macular ganglion cell layer volume measured on Optical Coherence Tomography (mm^3^).

When evaluating those who were asymptomatic or symptomatic at presentation, visual acuity (LogMAR) (Figure 3A), HVF mean deviation (Figure 3B) and OCT imaging measures (Figure 4A–C) did not significantly differ (Table 3). Their progression over time was comparable between the groups (Table 3, Figures 3A,D, 4A–C). The visual outcomes were good for both groups.

**TABLE 3:** Baseline and trajectory for visual and headache outcomes according to symptom status at diagnosis calculated by regression modelling.

	Baseline estimate	Change per month
LogMAR visual acuity, logunits		
Asymptomatic	−0.0275 (95% CI: −0.1201, 0.0652)	−0.0008 logunits/month (95% CI: −0.003, 0.0015)
Symptomatic	0.0365 (95% CI: 0.0037, 0.0692)	−0.0016 logunits/month (95% CI: −0.0029, −0.0003)
Humphrey visual field perimetric mean deviation, dB		
Asymptomatic	−2.1358 (95% CI: −4.5059, 0.2343)	0.0553 dB/month (95% CI: −0.0719, 0.1826)
Symptomatic	−3.4023 (95% CI: −4.1677, −2.6368)	0.1065 dB/month (95% CI: 0.0481, 0.165)
Global peripapillary retinal nerve fibre layer, μm		
Asymptomatic	140.52 (95% CI: 110.82, 170.21)	−1.81 μm/month (95% CI: −3.06, −0.56)
Symptomatic	135.68 (95% CI: 125.65, 145.72)	−1.49 μm/month (95% CI: −2.15, −0.82)
Global peripapillary total retinal thickness, μm		
Asymptomatic	373.69 (95% CI: 332.64, 414.73)	‐ 2.98 μm/month (95% CI: −4.45, −1.52)
Symptomatic	362.81 (95% CI: 348.92, 376.71)	−2.23 μm/month (95% CI: −2.99, −1.48)
Macular ganglion cell layer volume, mm^3^		
Asymptomatic	0.4514 (95% CI: 0.4251, 0.4778)	−0.0006 mm^3^/month (95% CI: −0.001, −0.0002)
Symptomatic	0.442 (95% CI: 0.4331, 0.4508)	−0.0003 mm^3^/month (95% CI: −0.0005, −0.0001)
Headache frequency (days/month)		
Asymptomatic	13.6 (95% CI: 8.06, 19.14)	0.06 units/month (95% CI: −0.36, 0.47)
Symptomatic	20.07 (95% CI: 18.13, 22.01)	−0.12 units/month (95% CI: −0.3, 0.06)
Migraine‐like headache frequency (days/month)		
Asymptomatic	3.6 (95% CI: −1.4, 8.59)	0.18 units/month (95% CI: −0.21, 0.58)
Symptomatic	9.11 (95% CI: 7.29, 10.92)	−0.03 units/month (95% CI: −0.2, 0.13)
Headache severity (0–10 scale)		
Asymptomatic	6.2393 (95% CI: 4.4749, 8.0037)	0.0178 units/month (95% CI: −0.0973, 0.133)
Symptomatic	6.5451 (95% CI: 6.0147, 7.0755)	−0.0131 units/month (95% CI: −0.0606, 0.0345)
HIT‐6 (score 36–78)		
Asymptomatic	59.77 (95% CI: 52.83, 66.7)	0.28 units/month (95% CI: −0.06, 0.63)
Symptomatic	61.33 (95% CI: 59.1, 63.55)	−0.04 units/month (95% CI: −0.18, 0.11)

Disease duration was the main influencing factor in determining baseline visual function in the asymptomatic group. Duration of disease influenced the baseline (time of database entry) estimates of VA, RNFL, and TRT [VA: increase of 0.00095 logunits/month (95% CI: 0.000171–0.001729); RNFL: decrease of 0.37 μm/month (95% CI: −0.61, −0.13); TRT decrease of 0.3307 μm/month (95% CI: −0.5717, −0.0897)], reflecting a slightly worse visual acuity and reduced OCT parameters the longer the disease duration. However, the trajectory following initial visit was not significantly influenced by disease duration, as only VA showed a small worsening of 0.00005 logunits/month (95% CI: 0.000003, 0.000096). Age had a small impact on macular GCL with thinner baseline GCL of 0.0011 mm^3^/year (95% CI: −0.0021, −0.0001). A personal or family history of migraine did not impact visual outcomes.

### 3.2 Headache outcomes

**FIGURE 5:**
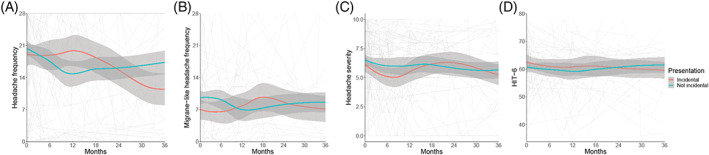
Longitudinal headache data categorised by symptoms at diagnosis, with Incidental papilloedema illustrated. LOESS smoothers added to show trends across the categories. (A) Headache frequency (days per month). (B) Migraine‐like headache frequency (days per month). (C) Headache mean severity of predominant headache (0–10 numerical rating scale). (D) Headache Impact Test 6 (HIT6) (quality of life measure score 36–78).

**FIGURE 6:**
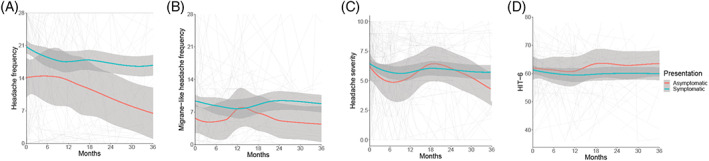
Longitudinal headache data categorised by symptoms at diagnosis, with Asymptomatic presentations illustrated. LOESS smoothers added to show trends across the categories. (A) Headache frequency (days per month). (B) Migraine‐like headache frequency (days per month). (C) Headache mean severity of predominant headache (0–10 numerical rating scale). (D) Headache Impact Test 6 (HIT6) (quality of life measure score 36–78)

Incidental presentations had a higher baseline headache and migraine‐like headache frequency (Figure 5A,B) than those with asymptomatic presentations (Figure 6A,B), although both were lower than the symptomatic group (Tables 2 and 3, Figure 6A,B). The headache severity and HIT‐6 quality‐of‐life measure were comparable between the groups (Tables 2 and 3, Figures 5C,D, 6C,D).

The asymptomatic patients had lower headache and migraine‐like headache frequency at the baseline visit compared to the symptomatic cohort (Table 3, Figure 6A,B), as would be expected. Of note, headache and migraine‐like headache frequency at baseline (time of enrolment to database) were not zero due to some patients developing headaches after papilloedema detection but prior to review. Over time of the 24 patients who became symptomatic during follow‐up, 23 reported headaches. Headache outcomes did not significantly change over time.

Daily headache at baseline visit was the main influential marker for worse outcomes [with a baseline headache frequency 17.31 days/month higher (95% CI: 15.11, 19.51)] but better reduction over time by 0.86 days/month (95% CI: −1.17, −0.56) and headache severity [baseline 3.02 units higher (95% CI: 2.15, 3.89) but trajectory better by 0.13 units/month (95% CI: −0.21, −0.04)]. A personal migraine history in this cohort was associated with a worse baseline headache disability (HIT‐6 score) by 3.13 units (95% CI: 0.09, 6.16) but for other measures only in univariate analysis, not when combined with daily headache at baseline.

## 4 DISCUSSION

This is the first prospective study to assess the long‐term outcomes of people with IIH who were asymptomatic at diagnosis with a comparison to those who were symptomatic. The comparable outcomes between these groups highlight that presenting without symptoms is not associated with either favourable or detrimental visual outcomes. Interestingly two‐thirds of these asymptomatic patients became symptomatic over the course of follow‐up, with almost all subsequently reporting headaches.

Incidental papilloedema was a common presentation with over one third of IIH patients presenting to this neuro‐ophthalmology service, however on direct questioning many had symptoms. This study's incidental papilloedema prevalence data are comparable to a study in North America (30.0%) but lower than what was reported in the United Kingdom (48.1%).^7^ 10% of people were completely asymptomatic at their first visit and this is higher than another retrospective study in adults, which showed 3.6% (5/139) of patients were asymptomatic.^16^ However their portion of asymptomatic presentations of IIH was similar to our proportion who remained asymptomatic through follow‐up (3.5%, 12/343). This highlights that most patients will develop symptoms through the course of their follow‐up and although managing asymptomatic IIH can be a concern for clinicians, it is actually a rare occurrence.

There were similar proportions of personal and family history of migraine in the asymptomatic and symptomatic groups which could indicate that the potential predisposition to headache may be similar. There was a higher prevalence of a prior migraine history in those cases who transitioned from no symptoms to suffering symptoms which could reflect the cyclical nature of migraine headaches.

The similar outcomes demonstrated in the incidental papilloedema group, and the asymptomatic group could indicate that those asymptomatic people are at the milder end of the IIH clinical spectrum. This is further supported by the lower surgical intervention rates in the asymptomatic cohort (Table 1). The higher diagnostic lumbar puncture opening pressure in the asymptomatic cohort highlights that the presence of symptoms may not be due to the absolute level of ICP. There may be many factors that determine whether an individual is experiencing symptoms.

Studying conditions of asymptomatic papilloedema is helpful as there remain many unanswered questions as to the development of papilloedema and the duration and severity required for permanent visual loss. This study of asymptomatic IIH may be interesting to clinicians, as one could consider those that are asymptomatic may present late which could confer worse outcomes but reassuringly this study's outcomes are similar to symptomatic IIH. On the other hand, those who are asymptomatic could be at the milder end of the disease spectrum, which does not appear to be the case here, with the evidenced similar outcomes. Parallels from this series could be drawn to astronauts undergoing long‐duration spaceflights that are relatively asymptomatic and through routine screening are found to meet the criteria to be diagnosed with spaceflight associated neuro‐ocular syndrome (SANS).^17^, ^18^ Therefore continued follow‐up of those with an asymptomatic presentation of papilloedema is warranted and should be managed as per symptomatic IIH.

The limitations of this study include the underestimation of asymptomatic prevalence due to only patients with a clear absence of all symptoms being included, and some symptoms may have been due to other conditions beyond IIH and therefore could have still been asymptomatic from their IIH. For example, a person with a prior history of migraine, may have had recurrence of migraine during follow‐up that was unrelated to their IIH diagnosis. This data is routinely collected clinical data and therefore standardised follow‐up intervals in the cohort were not possible, as follow‐up was dependent on disease status and papilloedema severity. Additionally, although the study aimed to capture all outcomes, patient preference meant some were missing. In future studies, comparison to a non‐IIH control group may be beneficial to enable better insights into whether changes noted in the asymptomatic cohort reflect the normal profile over time for a non‐IIH affected population, or whether this in fact is different between those groups due to underlying IIH pathophysiology in the absence of symptoms, for example, due to insulin resistance or hyperandrogenism associated with IIH.

Incidentally discovered papilloedema is a common occurrence, however, on direct questioning truly asymptomatic IIH has been found to be uncommon and becomes rare over the course of follow‐up as many begin to experience symptoms. Asymptomatic disease does not have either a beneficial or detrimental effect on visual outcomes as compared to symptomatic disease. Deterioration in papilloedema may be harder to detect in the asymptomatic population, and likely means this population needs routine eye examinations throughout the course of their lives.

## FUNDING INFORMATION

IIH:Life database is funded by the Healthcare Quality Improvement Partnership (HQIP) and by IIHUK registered patient charity (number 1143522) supported this work. Alexandra J. Sinclair has been funded during this study by Medical Research Council grant number MR/KO15184/1, National Institute of Health Research grant NIHR‐CS‐011‐028 (clinician scientist fellowship), Sir Jules Thorne Award for Biomedical Science.

## CONFLICT OF INTEREST STATEMENT

Mark Thaller and Victoria Homer report no conflicts. Susan P. Mollan reports consultancy fees (Invex Therapeutics; Neurodiem; Velux Foundation); advisory board fees (Gensight; Invex therapeutics; Janssen) and speaker fees (Heidelberg engineering; Chugai‐Roche Ltd; Allergan; Santen; Chiesi; and Santhera). Alexandra J. Sinclair reports personal fees from Invex therapeutics during the conduct of the study as well as share option and shareholdings, speaker fees (Novartis; Allergan; Teva UK) and consulting fees (Allergan; Chiesi; Novartis; Lundbeck).

## Data Availability

Professor Sinclair takes full responsibility for the data, the analyses and interpretation, and the conduct of the research. She has full access to all the data; and has the right to publish all data separate and apart from any sponsor. Proposals for data access should be made to the corresponding author. Reasonable scientifically sound proposals, from appropriately qualified research groups, will provide data beginning 12 months and ending 3 years after the publication of this article to researchers whose proposed use of the data are approved by the corresponding author. Requesters will need to sign a data access agreement, which will cover the terms and conditions of the release of data and will include publication requirements, authorship, acknowledgements and obligations for the responsible use of data.

## References

[1] Mollan SP , Davies B , Silver NC , et al. Idiopathic intracranial hypertension: consensus guidelines on management. J Neurol Neurosurg Psychiatry. 2018;89(10):1088‐1100. doi:10.1136/jnnp-2017-317440 29903905 PMC6166610

[2] Friedman DI , Liu GT , Digre KB . Revised diagnostic criteria for the pseudotumor cerebri syndrome in adults and children. Neurology. 2013;81(13):1159‐1165. doi:10.1212/WNL.0b013e3182a55f17 23966248

[3] Mollan SP , Virdee JS , Bilton EJ , Thaller M , Krishan A , Sinclair AJ . Headache for ophthalmologists: current advances in headache understanding and management. Eye (Lond). 2021;35(6):1574‐1586. doi:10.1038/s41433-021-01421-4 33580185 PMC8169696

[4] Thaller M , Homer V , Hyder Y , et al. The idiopathic intracranial hypertension prospective cohort study: evaluation of prognostic factors and outcomes. J Neurol. 2022;270:851‐863. doi:10.1007/s00415-022-11402-6 36242625 PMC9886634

[5] Mollan SP , Grech O , Alimajstorovic Z , Wakerley BR , Sinclair AJ . New horizons for idiopathic intracranial hypertension: advances and challenges. Br Med Bull. 2020;136(1):118‐126. doi:10.1093/bmb/ldaa034 33200788

[6] Mollan SP , Grech O , Sinclair AJ . Headache attributed to idiopathic intracranial hypertension and persistent post‐idiopathic intracranial hypertension headache: a narrative review. Headache. 2021;61(6):808‐816. doi:10.1111/head.14125 34106464

[7] Blanch RJ , Vasseneix C , Liczkowski A , et al. Differing presenting features of idiopathic intracranial hypertension in the UK and US. Eye (Lond). 2019;33(6):1014‐1019. doi:10.1038/s41433-019-0359-5 30783258 PMC6662244

[8] Kabanovski A , Wong JCY , Margolin EA , Micieli JA . Magnetic resonance or computed tomography venography in the evaluation of young overweight women with papilledema. Eye (Lond). 2021;35(8):2241‐2245. doi:10.1038/s41433-020-01242-x 33106608 PMC8302580

[9] Gondi KT , Chen KS , Gratton SM . Asymptomatic versus symptomatic idiopathic intracranial hypertension in children. J Child Neurol. 2019;34(12):751‐756. doi:10.1177/0883073819858455 31259642

[10] Lyons HS , Mollan SLP , Liu GT , et al. Different characteristics of pre‐pubertal and post‐pubertal idiopathic intracranial hypertension: a narrative review. Neuro‐Ophthalmology. 2022;47:1‐12. doi:10.1080/01658107.2022.2153874 36891406 PMC9988343

[11] Mollan SP , Aguiar M , Evison F , Frew E , Sinclair AJ . The expanding burden of idiopathic intracranial hypertension. Eye. 2019;33(3):478‐485. 30356129 10.1038/s41433-018-0238-5 PMC6460708

[12] Mollan SP , Tahrani AA , Sinclair AJ . The potentially modifiable risk factor in idiopathic intracranial hypertension: body weight. Neurol Clin Prac. 2021;11:e504‐e507. doi:10.1212/cpj.0000000000001063 PMC8382420 34484948

[13] Mollan SP , Mytton J , Tsermoulas G , Sinclair AJ . Idiopathic intracranial hypertension: evaluation of admissions and emergency readmissions through the hospital episode statistic dataset between 2002–2020. Life. 2021;11(5):417. doi:10.3390/life11050417 34063037 PMC8148005

[14] Krispel CM , Keltner JL , Smith W , Chu DG , Ali MR . Undiagnosed papilledema in a morbidly obese patient population: a prospective study. J Neuroophthalmol. 2011;31(4):310‐315. doi:10.1097/WNO.0b013e3182269910 21799447

[15] Bates DMM , Bolker B , Walker S . Fitting linear mixed‐effects models using lme4. J Stat Softw. 2015;67(1):1‐48. doi:10.18637/jss.v067.i01

[16] Rohani N , Foroozan R . Clinical course of asymptomatic patients with papilledema from idiopathic intracranial hypertension. Can J Ophthalmol. 2022;S0008‐4182(22)00058‐8. doi:10.1016/j.jcjo.2022.02.014 35304137

[17] Laurie SS , Lee SMC , Macias BR , et al. Optic disc edema and choroidal engorgement in astronauts during spaceflight and individuals exposed to bed rest. JAMA Ophthalmol. 2020;138(2):165‐172. doi:10.1001/jamaophthalmol.2019.5261 31876939 PMC6990717

[18] Scott RA , Tarver WJ , Brunstetter TJ , Urquieta E . Optic nerve tortuosity on earth and in space. Aerosp Med Hum Perform. 2020;91(2):91‐97. doi:10.3357/AMHP.5406.2020 31980047

